# Cross-cultural translation and modification of the revised oral assessment guide for oral health assessment by non-dentists

**DOI:** 10.1038/s41405-023-00168-2

**Published:** 2023-09-12

**Authors:** Nareudee Limpuangthip, Orapin Komin, Jumphitta Chaichaowarat, Patthamaporn Phumkor

**Affiliations:** 1 Department of Prosthodontics, Faculty of Dentistry, Chulalongkorn University, Bangkok, Thailand; 2 Dental Department, Maharat Hospital, Phra Nakhon Si Ayutthaya, Thailand; 3 Dental Department, Sungnoen Hospital, Nakornratchasima, Thailand

**Keywords:** Oral conditions, Dental conditions

## Abstract

**Objectives:**

This study aimed to conduct a cross-cultural translation of the revised oral assessment guide (ROAG) into Thai language and to modify the tool to increase its validity and reliability.

**Materials and methods:**

The present study was a cross-sectional design conducted in dental and hospitalized patients, and community-dwelling people. The original English-version of the ROAG was translated into Thai, which was evaluated for validity and reliability. The tool was then revised to develop the modified ROAG for non-dentist (ndROAG) comprising 9 oral assessment categories with a three-level response; healthy, mild, and severe alteration. The criterion validity of the ndROAG was tested in 82 adult and older participants, and 46 non-dentists comprising dental assistants, dental hygienists, community health volunteers, and nurses, using a calibrated dentist as the reference standard. The ndROAG was translated back into an English version. The criterion validity was evaluated using weighted Kappa (*K*_w_) and intraclass correlation coefficient (ICC). Internal consistency was determined using Cronbach alpha. The three-level response was dichotomized into healthy and changed to determine the sensitivity and specificity.

**Results:**

The *K*_w_ values, ICC, and Cronbach alpha values of the ndROAG were higher than those of the pre-test ROAG. The sensitivity of the ndROAG in identifying the healthy and changed state ranged from 57.1 to 100.0% with the lowest value in the saliva category, whereas the specificity ranged from 90.9–100.0%.

**Conclusion:**

The original ROAG was translated and revised into the ndROAG with improved validity and reliability. The ndROAG can be used by non-dentists to assess the oral health of adult and older individuals to detect oral changes, which includes self-care instructions and patient referral guidance.

## Introduction

The global aging population is increasing, leading to economic and social consequences such as a higher burden of systemic diseases and dependency [1]. Many older and dependent individuals face barriers to accessing oral health care and denture service due to socioeconomic limitations and physical or psychological impairments, resulting in infrequent dental visits [2, 3]. A simple oral health screening by health care personnel could enhance oral health care accessibility for community-dwelling people and hospitalized individuals, improving their overall oral health and well-being.

The dental personnel comprise dentists, dental assistants (DAs), and dental hygienists (DHs). DAs are responsible for chair-side assistance without performing dental treatment, whereas DHs can also provide simple dental treatments, such as scaling, and fluoride and sealant application. In Thailand, community health volunteers (CHV) work as primary health care providers at the community level, and are at the forefront in dealing with health care problems [3, 4]. CHVs receive training on basic medical care skills, and work as health information providers who take care of their own families and their neighbors [5]. Therefore, cooperation between dentists and other health care personnel are important for screening oral health problems, early detection, and controlling disease progression [6].

Several tools are available for non-dentists to assess oral health. A systematic review demonstrated that the revised oral assessment guide (ROAG), proposed by Andersson et al., is one of the most complete oral health assessment tools [7]. The tool is for non-dental personnel, such as nurses and caregivers, to determine the oral health problems of older or dependent people. The original Oral Assessment Guide (OAG) was developed by Eilers et al. to evaluate the oral health status of patients undergoing bone marrow transplantation [8]. The OAG was translated into Swedish and slightly modified by Andersson et al. to evaluate patients with hematological malignancies who underwent chemotherapy treatment [9]. In 2002, the OAG was revised into the ROAG, and firstly used in older patients residing in a rehabilitation ward by nurses [7]. The OAG modifications were made after a review of the literature, followed by suggestions from an expert panel.

The ROAG has been translated into several languages, such as Portuguese, German, and Swedish, and been used by nurses, CHVs, physicians, and caregivers to assess the oral health of older people [10–13]. Furthermore, modifications have been made to the ROAG to tailor its use for specific populations, including intensive care patients [14] and individuals undergoing chemotherapy [15, 16]. These adaptations involved the removal or addition of specific categories to enhance its applicability in these specialized contexts [14, 15]. However, the ROAG should be modified to include self-care instructions and improve its ability to identify dental treatment needs, particularly in the teeth and denture categories. Therefore, the objectives of this study were to conduct a cross-cultural translation of the ROAG into Thai language and to modify the tool to increase its validity and reliability.

## Materials and methods

The present study was a cross-sectional study design, conducted from January 2022 to November 2022. The protocol was approved by the Human Research Ethics Committee of the Faculty of Dentistry Chulalongkorn University (HREC-DCU 2020-103). The participants were adult and older individuals (40 years old and above), and non-dentists comprising DAs, DHs, CHVs, and nurses. The exclusion criteria were the patients who declined to receive an oral health assessment based on the ROAG, and the non-dentists who were unwilling to perform an ROAG assessment. The participants signed the written informed consent prior to study participation. For bedridden patients or those with dexterity limitations, their caregiver provided informed consent on their behalf.

**Fig. 1:**
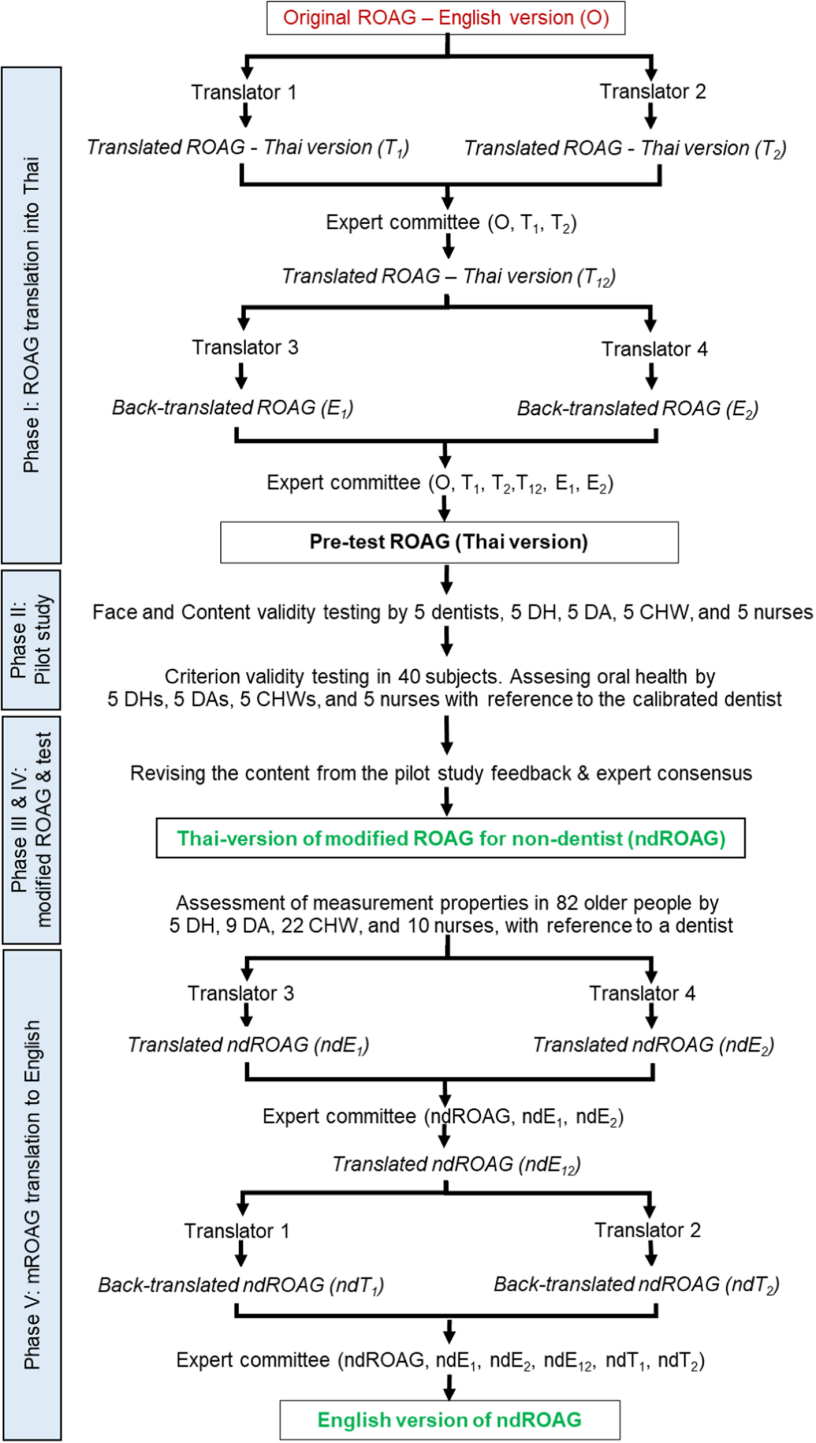
Flow diagram of the study. CHV community health volunteer, DA dental assistant, DH dental hygienist, ROAG revised oral assessment guide, O and E English-translated original ROAG, T Thai-translated original ROAG, mROAG modified version of the ROAG, ndE English-translated non-dentist ROAG, ndT Thai-translated non-dentist ROAG, ndROAG non-dentist ROAG

The development of the modified ROAG consisted of five phases (Fig. 1).

Phase I: Translation of the ROAG into the Thai-version

Phase II: Pilot study for the pre-test ROAG (Thai version) assessment

Phase III: Modification of the ROAG for non-dentists (ndROAG)

Phase IV: Assessment of the ndROAG

Phase V: Translation of the ndROAG into an English version

The participants who enrolled in Phase II and IV were patients, dental personnel, and health care workers from four settings.

Setting 1: DAs and dental patients who received prosthetic treatment at the Prosthodontic clinic, Faculty of Dentistry, Chulalongkorn University, Bangkok, Thailand,

Setting 2: DHs and dental patients who received dental treatment at the dental clinic at Maharat Hospital, Maharat District, Phra Nakhon Si Ayutthaya Province, Thailand,

Setting 3: Nurses and hospitalized patients in the Maharat Hospital, and

Setting 4: CHVs and community-dwelling dependent adults and older people who lived in Maharat District.

### Phase I. Translation of the ROAG into Thai-version

The original ROAG comprised 8 oral health assessment categories; voice, lips, mucous membrane, tongue, gums, teeth/denture, saliva, and swallowing. The scores for each category ranged from 0 (healthy) to 2 (severe alteration). The scores for each numerical and descriptive rating were added, giving a range between 0 (healthy) and 16 (severe alteration). The ROAG allows the assessor to determine whether the patient requires self-care only or should be referred to dentist or physician [7]. The translation process of the original English-version of ROAG to Thai was performed according to the WHO guidelines of the process of translation and adaptation of instruments [17], consisting of forward and backward translations, and expert panel consensus. After any discrepancies were discussed, a pre-test Thai-version of the ROAG tool was developed (Supplementary Table 1).

### Phase II: Pilot study for assessing the pre-test ROAG (Thai version)

The pre-test ROAG (Thai version) was administered to the participants in the target population, i.e., non-dentists, to assess the content and face validity. To evaluate content validity, 10 dentists assessed the content of the overall and each pre-test ROAG (Thai version) item based on a four-point Likert scale: very suitable, relatively suitable, needs slight modification, and needs extensive modifications. They also provided suggestions if any modifications were necessary to make a content suitable. Face validity was evaluated by 5 dentists and 20 non-dentists, comprising 5 DAs, 5 DHs, 5 CHVs, and 5 nurses. They gave responses whether the description of each item was clearly understood using a dichotomous scale (clearly understood or unclear), and provided suggestions if any rephrasing was necessary. The items with more than 20% of the participants answering “unclear” were considered as unclear.

The 20 non-dentists enrolled in a three-hour training program prior to assessing the ROAG in the adult and older participants. The training tools were photographs and audio files prepared in a Power Point presentation, showing different oral health conditions according to the ROAG. A speech researcher assisted in the audio selection for normal voice, slightly, or severely changed. Using a dentist as the reference, the trained non-dentists had to achieve an 80% weighted Kappa (K_w_) prior to performing the oral health assessment of the patients.

The inter-examiner agreement of the Thai-version of pre-test ROAG between two dentists (J.C. and P.P.) was evaluated in 40 patients (range 45–89 years old) from the four settings who were randomly selected from the hospital and community records. The criterion validity of the Thai-version of pre-test ROAG was evaluated by the 20 non-dentists with reference to the calibrated dentist (J.C.) who was considered as the reference standard. The unclear descriptions and inconsistencies between the dentists and non-dentists were discussed directly after each patient’s examination.

### Phase III: Modification of the ROAG

The results and comments obtained from phase II were discussed by nine experts, comprising five dentists, and one of the DAs, DHs, CHVs and nurses. The Thai-version of ROAG was then revised, and the modified ROAG for non-dentist (ndROAG) was developed (Supplementary Table 2). Modifications were made on the assessment methods, the descriptions in each category, self-care instructions, and patient referral. The teeth and denture were separated into two categories, resulting in nine categories. Similar to the original ROAG, the ndROAG had a three-level response for each category; healthy, mild alteration, and severe alteration. The scores for each category ranged from 0 (healthy) to 2 (severe alteration), giving the total score ranged from 0 (healthy) to 18 (severe alteration). If any category falls under the severe alteration level, it serves as an indication for the patient’s referral. The self-care instructions were established by expert consensus comprising two physicians, nurses, prosthodontists, and general dentists.

### Phase IV. Assessment of the ndROAG

This phase was performed to establish the full measurement properties of the ndROAG tool. Similar to the procedures of the ROAG-T, the face and content validity was evaluated by 10 dentists and 20 non-dentists. The criterion validity of the ndROAG was tested by the 46 non-dentists (9 DAs, 5 DHs, 22 CHVs, and 10 nurses) in 82 adult and older patients in the four study settings with 1–2 patients per non-dentist. The inter-examiner reliability of the ndROAG between two dentists (J.C. and P.P.) was evaluated in 15 patients and community-dwelling individuals who did not participate in the final test.

The 46 non-dentists enrolled in a three-hour training program prior to assessing the ndROAG in the adult and older patients, separated into individual sessions for the DAs, DHs, and nurses, and CHVs. The training tools were similar to phase II, except for the assessment descriptions and the oral structure photographs. Using the dentist as the reference, the trained non-dentists had to achieve an 80% K_w_ prior to performing the oral health assessment.

Oral health was assessed in 82 adult and older patients from the four settings who were randomly selected from hospital and community records. The criterion validity of the ndROAG was evaluated by the 46 non-dentists with reference to the calibrated dentist (J.C.) who was considered as the reference standard. One week later, the 46 non-dentists reevaluated the oral health in the same 46 participants at the same examination time to determine their intra-examiner reliability.

### Phase V: Translation of the ndROAG into English version

Similar to phase I, the forward-backward translation was performed to create an English version of the ndROAG, and a pre-test English-version of the ndROAG was developed (Table 1). The oral assessment aspects in each ndROAG category are summarized in Table 2.

**Table 1:** The modified ROAG for non-dentist (ndROAG) tool.

Category	Evaluation method	Score and descriptions			
		0 (Normal)	1 (Oral and denture care by the patient or caregiver)		2 (Dentist referral)
			Character	Self-care instruction	
1. Voice	Converse with patient and/or ask caregiver	Normal	Rasping, stuttering	Observe	Difficulty talking, voiceless
2. Lip (external surface)	Observe	Pink and smooth	Dry or cracked, and/or angular cheilitis	Frequently sip water and apply a lubricant	Abrasion or bleeding
3. Mucous membrane (inner lip, buccal mucosa and palate)	Observe use a light and finger palpation	Pink and moist	Dry, friction between the finger and mucosa and/or change in color, red, blue-red or white	Frequently sip water and observe	Very red, or thick, white coating Blisters or ulceration with or without bleeding
4. Tongue	Observe use light and finger with a gauze pad/clean fabric to hold and flip the tongue Rt-Lt and up-down	Pink, moist and papillae present	Dry, fissure tongue or white coating that can be brush/wipe-off	Frequently sip water and brush with a soft toothbrush twice a day	Very thick white coating that cannot be brushed/wiped-off; Intense red or white color; Blister or ulceration
5. Gum	Observe Use a light and finger palpation	Pink and firm around gingival margin No edematous	Edematous and/or red around gingival margin	Brush with a soft toothbrush and fluoride toothpaste twice a day	Generalized red edematous Blister or pus Bleeding easily under palpation
6. Teeth	Observe Use a light and finger touch and/or ask patient	Clean, No debris	Localize plaque or debris; Broken tooth that can function	Brush with a soft toothbrush and fluoride toothpaste twice a day	Generalized plaque or debris; Decayed tooth that traumatizes mucosa; Retained root; Very mobile tooth; Toothache that causes trouble in food chewing
7. Denture	Observe when denture in place and taken off and/or ask patient/caregiver	Clean and not damaged	Localize plaque or debris that can be brush/wipe-off; Damaged denture that can be function	Brush with a soft toothbrush and liquid soap or dishwashing liquid	Plaque or debris that cannot be brushed/wiped-off; Damaged denture that traumatize mucosa; Denture dislodges when chewing/speaking
8. Saliva	Finger moves along buccal mucosa	No friction	Some friction between the finger and mucosa	Frequently sip water	Significantly increased friction, the finger adheres to the mucosa
9. Swallow	Ask the patient to swallow, Observe, Ask the patient/caregiver	Normal swallowing	Some pain or difficulty on swallowing; Occasionally aspirate	Monitor while eating, sit upright while eating	Unable to swallow; Unable to be fed orally

The authors thank Dr Eilers for her role in developing the OAG and guiding translation into other languages for use worldwide.

**Table 2:** Oral assessment aspects in each ndROAG category (underlined indicates ‘not being assessed’ in the original ROAG).

Category	Evaluation aspects
1. Voice	- rasping - stuttering - difficulty talking - voiceless
2. Lips	- moistness - abrasion - bleeding
3. Mucous membrane	- moistness - color, white coating - blister - ulcer, bleeding
4. Tongue	- moistness - color - papilla - white coating that can be wiped-off - blister - ulcer
5. Gum -	- color - edema - blister, pus - bleeding
6. Teeth -	- plaque, debris - broken part - retained root - mobility - pain
7. Denture	- plaque, debris - broken part - dislodges when in function
8. Saliva	friction
9. Swallow	**-** difficulty swallow - pain - aspiration - tube/enteral/parenteral feeding

### Data analysis

The data were analyzed using SPSS version 29.0 for Windows (IBM, NY, USA). Because there is no agreed upon standard for oral health assessment, the calibrated dentist was the reference standard for the non-dentists’ examination. The criterion validity was evaluated using *K*_w_ statistics for the three-level responses and intraclass correlation coefficient (ICC) for the continuous summation score. The *K*_w_ values and their interpretations are: ≤0.2, poor; 0.21–0.40, weak; 0.41–0.60, moderate; 0.61–0.80, good; 0.81– 1.00, excellent The ICC interpretations are: <0.50, poor; 0.50–0.75, moderate; 0.75–0.90, good; and >0.90, excellent agreement [18]. The internal consistency of the pre-test ROAG and ndROAG was calculated as Cronbach’s alpha; a higher value indicated a higher correlation of the multiple categories within the tool [19]. The three-level response in the ndROAG was dichotomized into healthy and changed (mild or severe alteration), and the sensitivity and specificity were calculated for each category.

## Results

The adult and older patients in phase II (Thai-version of pre-test ROAG) and phase IV (ndROAG) were 80.0% and 84.1% female, with a mean age of 63.1 ± 10.5 years old (range 46–85 years old) and 66.1 ± 13.7 years old (range 40–91 years old), respectively. The mean age of the 46 non-dentists in phase IV was 63.1 ± 10.5 years old (range 24–70 years old) whose working experience ranged from 2–25 years. The inter-examiner reliability between the two dentists for each category and overall pre-test ROAG-T and ndROAG was 90–99% *K*_w_.

Based on the *K*_w_ values, the criterion validity of each pre-test ROAG-T category between the non-dentists and the calibrated dentist was poor to moderate, while that of the ndROAG was moderate to excellent agreement. In addition, the ICC values for the overall pre-test ROAG-T and ndROAG indicated poor and excellent agreement, respectively (Table 3). The Cronbach alpha value of the ndROAG was 12% higher than that of the Thai-version of pre-test ROAG. The sensitivity of the ndROAG in identifying the healthy and changed state ranged from 57.1 to 100.0% with the lowest value in the saliva category, whereas the specificity ranged from 90.9–100.0% (Table 4). The *K*_w_ for the intra-examiner reliability ranged from 0.75 to 0.99, with the lowest value in the saliva category. The mean time taken by the CHVs to perform each assessment for the ndROAG was 11 min (range 5–15 min).

**Table 3:** Weighted Kappa, intraclass correlation coefficient, and Cronbach alpha values of the Thai-version of pre-test ROAG and ndROAG determined by non-dentists using a dentist as a reference standard.

Categories	Pre-test ROAG (*N* = 40)		ndROAG (*N* = 82)	
	Weighted Kappa	95% CI	Weighted Kappa	95% CI
1. Voice	0.732	(0.48, 0.99)	1.00	(1.00, 1.00)
2. Lips	0.388	(0.13, 0.64)	0.82	(0.58, 1.06)
3. Mucous membrane	0.423	(0.06, 0.79)	0.88	(0.71, 1.05)
4. Tongue	0.402	(0.11, 0.69)	0.86	(0.75, 0.96)
5. Gums	0.302	(0.07, 0.54)	0.89	(0.80, 0.98)
6. Teeth	0.35	(0.08, 0.62)	0.84	(0.73, 0.94)
7. Dentures			0.89	(0.76, 1.02)
8. Saliva	0.10	(0.02, 0.39)	0.71	(0.40, 1.02)
9. Swallow	0.69	(0.36, 1.01)	1.00	(1.00, 1.00)
*Total score (ICC)*	0.69	(0.48, 0.82)	0.91	(0.86, 0.94)
Cronbach alpha	0.599^a^		0.671	

^a^Voice, saliva and swallow variables has zero variance and were removed from the scale.

**Table 4:** Sensitivity and specificity of the ndROAG determined by non-dentists using a dentist as a reference standard.

Categories	Sensitivity	Specificity
1. Voice	100.0	100.00
2. Lips	97.40	100.0
3. Mucous membrane	75.00	100.0
4. Tongue	85.70	100.0
5. Gums	84.8	100.0
6. Teeth (*n* = 67)	97.8	90.90
7. Dentures (*n* = 46)	87.5	100.0
8. Saliva	57.1	100.0
9. Swallow	100.0	100.0

## Discussion

In this study, the ROAG was translated into Thai and modified to be used as an oral health assessment tool for dental and non-dental professional use in clinical and community settings. Modifications were made on the clinical evaluation equipment, assessment methods and criteria, and patient referral, and included self-care instruction. The ndROAG demonstrated increased validity when an oral health assessment was performed by non-dentists using the dentist as the reference standard.

Previous studies have proposed various modified versions of the ROAG to improve efficiency and enhance sensitivity in detecting specific oral changes. Ribeiro et al. modified the ROAG by excluding the teeth category and separating the mucous membrane into labial mucosa and buccal mucosa or palate categories [15]. This modification aimed to evaluate changes in the oral mucosa resulting from antineoplastic treatment involving chemotherapeutics [15, 16]. Similarly, Doi et al. simplified the ROAG tool, focusing on three categories: tongue, mucous membranes, and saliva. Their objective was to assess thirst perception and dry mouth in patients receiving intensive care [14]. In contrast, our study expanded the number of ROAG categories by separating teeth and denture assessments. Although this may increase the evaluation time, the ndROAG allows for the detection teeth and denture-related problems in general adults and older individuals.

The original ROAG comprises an intra- and extra oral examination using a light and mouth mirror. However, the mouth mirror for evaluating saliva has been removed from the ndROAG because it is not applicable for home use in a community setting. Using the mirror has been replaced with using fingers and a gauze pad. Moreover, patient and caregiver responses are included when examining the voice, swallowing, teeth, and denture. This is because an abnormal voice and swallowing may not be detected at the time of evaluation, however, these can be detected by their changes reported by the older people or their caregivers. A raspy voice in some older people could be due to increased age rather than an abnormal change. To assess the patient’s swallowing ability, the ndROAG adds aspiration as a sign of mild alteration because it is one of the common signs and symptoms of dysphagia, which can lead to malnutrition, aspiration pneumonia, and mortality [20, 21]. A caregiver response is required, particularly for patients with a nasogastric tube. Adding self-reported evaluations by the patients and their caregiver to the professional evaluation allows for self-detected changes, and future application of the ndROAG in patients with cognitive impairment, such as dementia and Alzheimer’s disease.

Major changes were made to the teeth and denture category by separating them into two categories to identify problem sources and treatment needs. The original ROAG focused only on the presence of plaque on the teeth and denture, which does not accurately reflect the severity of the problem. In the ndROAG, descriptions for a broken tooth, tooth mobility, and a retained root were added to the teeth category. This was because our pilot study demonstrated that a broken tooth usually traumatizes the surrounding mucosa, and a retained root can be a source of odontogenic infection. Pain perception was also included because symptomatic teeth without any cavitation were reported in some older patients. In the denture category, additional criteria were denture chipping, because it can traumatize the oral mucosa, and an ill-fitting denture, which is one of the most common complaints among denture wearers [22, 23].

The gum category in the original ROAG considered only color change and bleeding. The results of our pilot study revealed that non-dentists usually identified color changes on the attached and unattached gingiva, and commented on the difficulty in consistently identifying the gingival color as pink or red. The ndROAG clarifies gingival inflammation by focusing on the gingival margin, and including gingival swelling and the presence of pus or exudate. These characteristics were included because they can be caused by local inflammation and some systemic medications [24, 25], which require dentist and physician consultations.

The ndROAG provides a detailed clarification of the mucosal locations in that the lip indicates its external surface, whereas the mucosa covers the inner lip, buccal mucosa, and palate. When oral dryness is present, it covers the tongue, mucosa, and saliva categories. The ndROAG requires using the fingers to move along the mucosa for saliva evaluation, to retract the lips for mucosal evaluation, and to lift and flip the tongue to evaluate its dorsum and sublingual aspects. The presence of a white coating in the original ROAG indicates a dentist referral, however, in the ndROAG, a white coating that can be wiped-off using a gauze pad remains in a mild alteration, and a dentist referral is indicated when it cannot be wiped-off. Although the evaluation method was modified, the saliva category demonstrated the lowest *K*_w_ score because it was difficult to differentiate between normal and slight change in friction using an individual’s tactile sense. This may be because an evaluation method that requires tactile sense is more subjective compared with those using an observation and patient-reported outcome.

The ndROAG was modified to include self-care instructions after the examination criteria for a non-dentist to promptly advise the patients and their caregivers if mild alteration is detected. The use of artificial saliva substitute was removed because the patients should have been diagnosed and the cause of decreased saliva identified prior to receiving optimal treatment and management [26]. In the original ROAG, the management of a severe alteration is somewhat confusing because some categories indicate a referral to a dentist and some to a physician, or to perform self-care. Thus, a severe alteration observed in any category of the ndROAG indicates a dentist referral, and allows them to decide whether a physician consultation is needed. We suggest that the categorical score for each individual category demonstrated efficacy in screening oral problems, whereas the continuous score would be more suitable for monitoring changes overtime.

The utilization of ndROAG is suggested as a screening tool for patients and non-dentists to facilitate early detection of oral problems, fostering self-awareness, and encouraging oral self-care, ultimately promoting improved oral health outcomes for patients. Although the original ROAG had been used only in older people [7, 10–13], the ndROAG can be used with adult and older patients. Using the ndROAG strengthens the capability of the primary healthcare setting by enabling community-dwelling people, community health worker, as well as non-dental personnel to detect and manage the initial stage of oral diseases. In the future, the DHs and CHVs should be a family caregiver who is responsible for providing individual oral care for dependent community-dwelling people and serve as a link between dentists and individual patients. By employing the ndROAG score, healthcare professionals can customize treatment plans according to individual patient needs. Overall, the global use of the ndROAG score can standardize oral health assessment, improve patient outcomes, promote interdisciplinary collaboration, and contribute to advancements in oral healthcare research and practices.

The present study has some limitations. Because this was a cross-sectional study design, the responsiveness or ability of the ndROAG to detect changes over time has not been evaluated. In the present study, the dentist plays an important role in ndROAG evaluation and referral, however, in practice, a multidisciplinary approach is required. Thus, the patients’ referral options may not be limited to dentists, but involve other health personnel that may vary among clinical and community settings. Additional studies are needed to confirm the validity of the ndROAG and its translation in English and other languages, and to extend its generalizability to other non-dentists and patients with functional limitations or those living in a residential home care. Training DHs and CHVs for screening oral disease and providing basic oral care is necessary to strengthen primary oral health care, and reduce the oral disease burden.

## Conclusion

The original ROAG was translated and revised into the ndROAG with improved validity and reliability. The ndROAG can be used by non-dentists to assess the oral health of adult and older individuals for early detection of oral changes and provide self-care instructions and patient referral.

### Supplementary information

Supplementary Information

## Data Availability

The data used in this study can be provided upon request to the corresponding author.
