# Supplementary material for: Cross-cultural translation and modification of the revised oral assessment guide for oral health assessment by non-dentists
Source: BDJ Open. 2023 Sep 12;9:42. doi: 10.1038/s41405-023-00168-2 (PMC10497511; doi:10.1038/s41405-023-00168-2)
Supplement: Supplementary file 1 — Supplementary Information [file 41405_2023_168_MOESM1_ESM.pdf]

Supplementary Table 1. The Thai-translated ROAG. The authors thank Dr Eilers for her role in developing the OAG and guiding translation into other languages for use worldwide

สิ่งที่ตรวจ	วิธีการตรวจ	ระดับคะแนนและคำอธิบาย			ขั้นตอน
		1 (ปกติ)	2	3	
**1. เสียง**	พูดคุยและฟังเสียงของผู้ป่วย	ปกติ	เสียงแหบ เสียงพูดติดขัด ไม่ต่อเนื่อง	พูดลำบาก เปล่งเสียงไม่ออก	ปรึกษาแพทย์
**2. ริมฝีปาก**	สังเกต	มีสีชมพู และเรียบ	แห้งหรือมีรอยแตก และ/หรือ มุมปากอักเสบ	เป็นแผล หรือมีเลือดออก	ปรึกษาแพทย์หรือทันตแพทย์
**3. เนื้อเยื่อในช่องปาก** (เมื่อถอดฟันปลอม)	สังเกตโดยใช้ไฟส่องและกระจกส่องปาก	มีสีชมพู และชุ่มชื้น	แห้ง และ/หรือ เปลี่ยนเป็นสีแดง สีแดงอมม่วง หรือสีขาว	มีสีแดงจัด<br>หรือมีคราบหนาสีขาวปกคลุม<br>มีตุ่มน้ำ<br>หรือเป็นแผลที่มีเลือดออกหรือไม่ก็ได้	ปรึกษาแพทย์หรือทันตแพทย์
**4. ลิ้น**	สังเกตโดยใช้ไฟส่องและกระจกส่องปาก	มีสีชมพู ชุ่มชื้น<br>และเห็นตุ่มลิ้น	แห้ง ไม่เห็นตุ่มลิ้น<br>หรือลิ้นเปลี่ยนเป็นสีแดงหรือสีขาว	มีคราบหนาสีขาวปกคลุม<br>มีตุ่มน้ำหรือมีแผล	ปรึกษาแพทย์หรือทันตแพทย์
**5. เหงือก**	สังเกตโดยใช้ไฟส่องและใช้นิ้วสัมผัส	มีสีชมพู<br>และขอบเหงือกแน่น	บวม และ/หรือมีสีแดง	เลือดออกง่ายเมื่อใช้นิ้วสัมผัส	ดูแลทำความสะอาดช่องปาก<br>และปรึกษาทันตแพทย์<br>หรือทันตบุคลากร
**6. ฟัน ฟันปลอม**	สังเกต<br>โดยใช้ไฟส่องและกระจกส่องปาก	สะอาด<br>ไม่มีสิ่งสกปรกหรือเศษอาหารตกค้าง	1) มีคราบ<br>สิ่งสกปรกหรือเศษอาหารบนตัวฟันหรือฟันปลอมบางบริเวณ<br>2) มีฟันผุ หรือฟันปลอมบิ่น แตกหัก	มีคราบ<br>สิ่งสกปรกหรือเศษอาหารโดยทั่วไปบนฟันหรือฟันปลอม	1)<br>ดูแลทำความสะอาดช่องปาก<br>2)<br>ปรึกษาทันตแพทย์<br>หรือทันตบุคลากร

สิ่งที่ตรวจ	วิธีการตรวจ	ระดับคะแนนและคำอธิบาย			ขั้นตอน
		1 (ปกติ)	2	3	
**7. น้ำลาย**	ใช้กระจกส่องปากลากตามแนวกระพุ้งแก้ม	ลากได้ลื่นตลอดแนวกระพุ้งแก้ม	รู้สึกฝืดเล็กน้อยขณะลาก<br>โดยกระจกส่องปากไม่ติดกับกระพุ้งแก้ม	กระจกส่องปากติดกับกระพุ้งแก้มขณะลาก	ดูแลทำความสะอาดช่องปาก<br>ใช้น้ำลายเทียม
**8. การกลืน**	ให้ผู้ป่วยกลืนน้ำลาย สังเกต และ/หรือ ซักถามผู้ป่วย	กลืนได้ปกติ	เจ็บขณะกลืน หรือกลืนลำบาก	กลืนไม่ได้เลย	ปรึกษาแพทย์

Supplementary Table 2. Thai-version of mROAG. The authors thank Dr Eilers for her role in developing the OAG and guiding translation into other languages for use worldwide

**สิ่งที่ตรวจ**	**วิธีการตรวจ**	**ระดับคะแนนและคำอธิบาย**			
		**0 (ปกติ)**	**1 (ดูแลสุขภาพช่องปาก/ฟันปลอมด้วยตัวเองหรือผู้ดูแล)**		**2 (พบทันตแพทย์)**
			**สิ่งที่ตรวจพบ**	**คำแนะนำ**	
**1. เสียง**	พูดคุยและฟังเสียงของผู้ป่วย และ/หรือถามผู้ดูแล	ปกติ	เสียงแหบ เสียงพูดติดขัด ไม่ต่อเนื่อง	สังเกตอาการ	พูดลำบาก เปล่งเสียงไม่ออก
**2. ริมฝีปากด้านนอก**	สังเกต	มีสีชมพู และเรียบ	แห้งหรือมีรอยแตกเป็นขุย หรือลอกเป็นแผ่น	จิบน้ำบ่อยๆ และใช้สารหล่อลื่นทาที่ริมฝีปาก	เป็นแผล หรือมีเลือดออก และ/หรือ มุมปากอักเสบ
**3. เนื้อเยื่อในช่องปาก** (กระพุ้งแก้ม ริมฝีปากด้านใน และเพดานปาก)	สังเกต โดยใช้ไฟส่องและใช้นิ้วสัมผัส	มีสีชมพู และชุ่มชื้น	แห้ง ฝืดเมื่อใช้นิ้วสัมผัส และ/หรือ เปลี่ยนเป็นสีแดง สีแดงอมม่วง หรือสีขาว	จิบน้ำบ่อยๆ และสังเกตอาการ	มีสีแดงจัด หรือมีคราบหนาสีขาวปกคลุม มีตุ่มน้ำ หรือเป็นแผลที่มีเลือดออกหรือไม่ก็ได้
**4. ลิ้น**	ใช้ผ้าก๊อซ หรือผ้าสะอาดจับปลายลิ้นพลิกซ้าย-ขวา และยกลิ้นขึ้น สังเกตโดยใช้ไฟส่อง	มีสีชมพู ชุ่มชื้น และเห็นตุ่มลิ้น	แห้งแตกเป็นร่อง หรือมีคราบปกคลุมที่แปรง/เช็ดออก	จิบน้ำบ่อยๆ และแปรงลิ้นวันละ 2 ครั้งด้วยแปรงสีฟันขนนุ่ม	มีคราบหนาปกคลุมที่แปรงหรือเช็ดไม่ออก ลิ้นเปลี่ยนเป็นสีแดงหรือสีขาว หรือมีตุ่มน้ำ/มีแผล
**5. เหงือก**	สังเกต โดยใช้ไฟส่องและใช้นิ้วสัมผัส	มีสีชมพู และขอบเหงือกแน่น ไม่บวมแดง	ขอบเหงือกบวม และ/หรือมีสีแดง	แปรงฟันด้วยแปรงสีฟันขนนุ่ม และยาสีฟันที่มีส่วนผ	เหงือกบวมแดงโดยทั่วไป มีตุ่มหนองหรือหนองไหล เลือดออกง่ายเมื่อใช้นิ้วสัมผัส

สิ่งที่ตรวจ	วิธีการตรวจ	ระดับคะแนนและคำอธิบาย			
		0<br>(ปกติ)	1<br>(ดูแลสุขภาพช่องปาก/ฟันปลอมด้วยตัวเองหรือผู้ดูแล)		2<br>(พบทันตแพทย์)
			สิ่งที่ตรวจพบ	คำแนะนำ	
				สมของฟลูออไรด์<br>วันละ 2 ครั้ง	
**6. ฟัน**	สังเกต<br>โดยใช้ไฟส่อง<br>และใช้นิ้วจับโยก<br>และ/หรือ<br>ซักถามผู้ป่วย	สะอาดโดยทั่วไป	มีคราบ<br>หรือเศษอาหารบนฟันบางตำแหน่ง<br>หรือ<br>ฟันบิ่นแตกแต่ยังใช้งานได้	แปรงฟันด้วยแปรงสีฟันขนนุ่ม<br>และยาสีฟันที่มีส่วนผสมของฟลูออไรด์<br>วันละ 2 ครั้ง	มีคราบ<br>หรือเศษอาหารบนฟันโดยทั่วไป<br>ฟันบิ่นแตกและบาดเนื้อเยื่อหรือมีรากฟันคงค้างที่ใช้งานไม่ได้<br>ฟันโยกอย่างมากเมื่อใช้นิ้วสัมผัส<br>หรือ ปวดฟันจนเคี้ยวอาหารไม่ได้
**7. ฟันปลอม**	สังเกตทั้งขณะใส่และถอดฟันปลอม<br>และ/หรือ<br>ซักถามผู้ป่วย/ผู้ดูแล	สะอาดโดยทั่วไป<br>และ ไม่แตกบิ่น	มีคราบ<br>หรือเศษอาหารบนฟันปลอมที่แปรง/เช็ดออก<br>หรือ<br>ฟันปลอมบิ่นแตกแต่ยังใช้งานได้	แปรงฟันด้วยแปรงสีฟันขนนุ่ม ร่วมกับน้ำสบู่<br>หรือน้ำยาล้างจาน	มีคราบ<br>หรือเศษอาหารบนฟันปลอมโดยทั่วไปที่แปรงไม่ออก<br>หรือ<br>ฟันปลอมบิ่นแตกและบาดเนื้อเยื่อ<br>หรือ<br>ฟันปลอมหลุดขณะเคี้ยวหรือพูด
**8. น้ำลาย**	ใช้นิ้วลากตามแนวกระพุ้งแก้ม	ลากได้ลื่นตลอดแนวกระพุ้งแก้ม	รู้สึกฝืดขณะลาก	จิบน้ำบ่อยๆ	นิ้วติดกระพุ้งแก้ม<br>ไม่สามารถลากได้
**9. การกลืน**	ให้ผู้ป่วยกลืนน้ำลาย<br>สังเกต<br>และ/หรือ<br>ซักถามผู้ป่วย/ผู้ดูแล	กลืนได้ปกติ	เจ็บ กลืนลำบาก<br>หรือสำลักเป็นบางครั้ง	-<br>เฝ้าระวังสำลักขณะกินอาหาร<br>-<br>กินอาหารในท่านั่งเสมอ	กลืนไม่ได้เลย<br>หรือกินอาหารทางปากไม่ได้
